# A Novel, Efficient Method to Isolate Chicken Primordial Germ Cells from Embryonic Blood Using Cell Culture Inserts

**DOI:** 10.3390/ani13243805

**Published:** 2023-12-09

**Authors:** Xia Zhang, Rui Xian, Yingxiao Fu, Yanyan Dai, Rui Peng

**Affiliations:** Key Laboratory of Bio-Resources and Eco-Environment, Ministry of Education, College of Life Sciences, Sichuan University, Chengdu 610064, China

**Keywords:** primordial germ cells, cell culture insert, feeder-free culture system

## Abstract

**Simple Summary:**

Primordial germ cells (PGCs) play a critical role in the preservation of poultry genetic resources and transgenic research, but there are significant efficiency issues with isolating PGCs from chicken embryonic blood, especially from a single embryo. In this study, we developed a simple and rapid method to isolate PGCs from single chicken embryonic blood. This method is based on the characteristics of PGCs found in the study. That is, when co-cultured with chicken embryonic fibroblasts (CEFs) of passages two to three, PGCs can migrate to the lower layer of CEF through pores smaller than their diameter, which is called cell culture inserts/CEF adhesion method. The PGCs isolated using this method retain their stem cell characteristics and migration ability, and exhibit good proliferation efficiency. These cells can be directly utilized for subsequent transgenic experiments or for the preservation of germplasm resources.

**Abstract:**

Primordial germ cells (PGCs) play a crucial role in preserving poultry genetic resources and conducting transgenic research. A system for the rapid isolation of PGCs from single chicken embryonic blood was established in this paper. We found that PGCs can migrate to the lower layer of chicken embryonic fibroblasts (CEFs) through pores smaller than their diameter, while blood cells cannot, when co-cultured with CEFs of passages two to three. Based on the characteristics of PGCs, we developed a new PGC isolation method (cell culture insert/CEF adhesion method) that utilizes a 3 μm cell culture insert and CEFs of passages two to three. Using this method, approximately 700 PGCs can be isolated from the blood of a single chicken embryo at Hamburger and Hamilton (H&H) stage 17 of development. The separation rate achieved was 87.5%, with a separation purity of 95%. The separation rate of this method was 41.4% higher than the common Percoll density gradient centrifugation method and 33.6% higher than lysis with ACK buffer. PGCs isolated from embryonic blood could proliferate 37-fold within 2 weeks when cultured in a feeder-free culture system. They also continued to express the *SSEA-1* and *DAZL* proteins and retained the ability to migrate in vivo. Overall, PGCs separated using cell culture inserts/CEF adhesion method retain their stem cell characteristics and migration ability. PGCs also exhibit good proliferation efficiency, making them suitable for subsequent transgenic experiments or genetic resource preservation.

## 1. Introduction

Avian species have unique reproductive structures and embryonic development processes, which make gene editing more challenging in birds. Primordial germ cells (PGCs) can differentiate into germ cells, which transmit the entire genetic information to the next generation. The successful isolation of PGCs lays the foundation for the conservation of avian genetic resources and transgenic operations [1,2,3]. Avian PGC shows a distinct migration pathway during embryo development. PGCs originate in the epiblast layer, are scattered throughout the vascular system, and circulate with the blood flow from Hamburger and Hamilton (HH) stage 13 to 17 [4]. They then migrate to the germinal ridges at HH stage 27. The number of PGCs in the blood is highest at HH stage 17 [5,6,7]. The unique migration pattern of avian PGCs facilitates their isolation from the bloodstream and germinal ridges. So far, the method of isolating PGCs from the chick embryo gonadal ridge or gonad is relatively mature. Compared to PGCs derived from blood, however, gonadal PGCs are at a relatively late developmental stage compared to PGCs from blood and have different gene expression patterns. Therefore, obtaining high-purity PGCs from blood is crucial for the study of the early development and migration of PGCs.

At present, there are several commonly used methods to isolate and purify avian PGCs from blood. The first method is density gradient centrifugation, which includes Ficoll density gradient centrifugation, Percoll density gradient centrifugation, and Nycodenz density gradient centrifugation. Although Ficoll density gradient centrifugation is effective at removing blood cells, it yields only a small number of highly pure PGCs. In addition, the Nycodenz density gradient centrifugation method requires the preparation of five Nycodenz solutions with different concentration gradients. These methods are relatively difficult to operate and have low isolation and purification efficiencies [8,9,10]. In order to simplify the centrifugation step, ACK lysis buffer is used to lyse blood cells. The lysed blood cells can then be removed by centrifugation. However, there are still many dead red blood cells mixed in with the isolated PGCs, and the purity of PGCs can only be improved after 7 days of culture or through secondary lysis. Additionally, it is unclear whether the lysates will affect the long-term culture of PGCs [11]. In general, 500 to 2000 PGCs can be obtained from 20 chicken embryos using the aforementioned methods. In addition, magnetic-activated cell sorting (MACS) and fluorescence-activated cell sorting (FACS) are also employed to isolate primordial germ cells (PGCs). Although the purity of PGCs is high, the separation efficiency is low. On average, only about 50 primordial germ cells (PGCs) can be obtained per sample, and the cost is also high [12,13]. The aforementioned isolation methods all require more than 10 donor samples and are not suitable for separating a small number of samples. This limitation hinders the preservation of rare poultry genetic resources or the transgenic manipulation of specific poultry breeds.

In this paper, a simple isolation method was developed based on the adhesion properties of PGCs to the chicken embryo fibroblasts (CEFs) of passages 2 to 3. This method improves the efficiency and effectiveness of isolating PGCs from a single chicken embryo. It is possible to provide technical support for obtaining transcriptomic data from PGCs in the early stage, specifically before the colonization of the gonadal ridge.

## 2. Materials and Methods

### 2.1. Collection of Chicken Embryonic Blood

Fertilized White Leghorn (WL) eggs were provided by Fuguangxing Animal Husbandry (Mianyang, China). Incubate the fresh eggs until they reach HH stage 17 (52–64 h). Sterilize the eggshell using 75% alcohol. In a biological safety cabinet, use a capillary glass needle to extract approximately 10 µL of chicken embryonic blood from the dorsal aorta. Transfer the extracted blood into a 24-well plate containing 500 µL of PGC medium. The experimental methods were approved by the Animal Use and Care Committee of the College of Life Sciences at Sichuan University.

### 2.2. Preparation of PGC Medium

The culture medium for PGCs was appropriately modified following the protocol of Collarini EJ [14]. The modified medium consisted of 55% KO-DMEM (Gibco, 10829-018, Waltham, MA, USA), 20% CEF-conditioned medium, 15% FBS (ExCell Bio, Shanghai, China), 5% chicken serum (VivaCell, Shanghai, China), 100× Embryo MAX^®^ Nucleosides (Millipore, Burlington, MA, USA), 100× MEM NEAA (Gibco, Waltham, MA, USA), 100× Glutamax (Gibco, Waltham, MA, USA), 100× penicillin-streptomycin-amphotericin B mixed solution (Solarbio, Beijing, China), 0.1 mmol/L β-ME (Sigma, Saint Louis, MO, USA), 1× B27 Supplement (Gibco, Waltham, MA, USA), 0.1 mg/mL heparin sodium (BBI, Shanghai, China), 6 ng/mL SCF (Novoprotein, Suzhou, China), 4 ng/mL bFGFb (Novoprotein, Suzhou, China), 25 ng/mL Activin-A (Novoprotein, Suzhou, China), LIF (Novoprotein, Suzhou, China), BMP4 (Proteintech, Wuhan, China), and TGF-β1 (Novoprotein, Suzhou, China). Adding a high proportion of FBS to the culture medium can promote the proliferation of PGCs, which may be due to the presence of various growth factors in fetal bovine serum. The preparation method for the CEF-conditioned medium involves spreading chicken embryonic fibroblasts in a 10 cm plastic Petri dish, adding 8 mL of DMEM containing 10% FBS, and collecting the culture medium after 48 h of culture. The culture medium from CEFs at passage numbers 2 to 5 was pooled and centrifuged at 1200 rpm for 3 min. The supernatant was then filtered through a 0.22 μm sterile filter and aliquoted before being stored at −20 °C.

### 2.3. Preparation of Feeder Cells

After an incubation for 9–11 days, the chicken embryos were dissected to remove the head, tail, limbs, and viscera. Then, the samples were digested with 0.25% trypsin-EDTA (Biosharp, Hefei, China) in a water bath at 37 °C for 15 min. The digestion was terminated by adding DMEM containing 10% FBS, and the residue was removed through filtration. After centrifugation, approximately 2 × 10^6^ cells were seeded in a 10 cm cell culture dish. After culturing cells overnight, rinse with PBS three times to remove the non-adherent cells from the upper layer, and continue culturing until the cell confluence reached 80%. CEF cells were treated with mitomycin C (Selleck, Shanghai, China) and then placed in a 24-well plate that had been coated with 0.1% gelatin. After CEFs attached to the wall, they could be used as feeder cells.

### 2.4. Isolation of PGCs

Percoll Density Gradient Centrifugation Method: Mix 9 parts of Percoll stock solution (Solarbio, Beijing, China) with 1 part of 1.5 M NaCl (*v*/*v*). Then, add a specific proportion of PBS to prepare Percoll solutions with concentrations of 12.5%, 25%, and 50%. Add 50%, 25%, and 12.5% Percoll solutions into a 15 mL centrifuge tube in sequence to prepare the Percoll concentration gradient solution. Then, carefully add chicken embryo blood onto the top layer. Centrifuge at 3000 rpm for 20 min at room temperature. After centrifugation, PGCs are located between the 25% and 50% solutions of the Percoll gradient. Transfer the solution containing PGCs and transfer it to a new 15 mL centrifuge tube. Then, add 6 mL of PBS to dilute it. After centrifugation at 2500 rpm for 10 min, PGCs precipitate at the bottom of the centrifuge tube. After washing with PBS twice, add 500 µL of PGC medium to resuspend the cells, and then transfer them to a 24-well plate for culture.

ACK Lysis Method: Chicken embryonic blood was centrifuged at 2500 rpm for 10 min, and the cell pellet was resuspended in 100 µL of PBS. Then, 900 µL of ACK lysis buffer (150 mM NH_4_Cl, 1 mM KHCO_3_, 0.001 mM EDTA) was added. After incubating the solution on ice for 30 min, centrifuge it at 2500 rpm at 4 °C for 10 min. The precipitate was resuspended in 1000 µL of ACK lysis buffer and incubated on ice for 15 min. After centrifugation at 2500 rpm for 10 min at 4 °C, the cell pellet was washed twice with PBS, resuspended by adding 500 µL of PGC medium, and transferred to a 24-well plate.

Cell culture insert/CEF adhesion method: Place cell culture inserts with different apertures into a 24-well plate containing feeder cells (CEFs). Then, add chicken embryonic blood into the cell culture inserts. PGCs and CEF cells were co-cultured using PGC culture medium. When PGCs migrate to the lower CEFs, remove the cell culture insert. Gently wash the PGCs attached to CEFs with PGC culture medium and then transfer them to a 24-well plate coated with 0.1% gelatin. After an overnight culture, detach the attached CEFs to obtain high-purity PGCs.

PKH67 staining: PGCs obtained using the cell culture insert/CEF adhesion method were washed 3 times with PBS and resuspended in 100 μL of staining workup (Solarbio, Beijing, China) at a concentration of approximately 10^4^/μL. The cells were incubated at 37 °C for 5 min and then at 4 °C for 15 min. After centrifugation, the supernatant was removed and the cells were collected. The cells were washed twice with PBS, resuspended in PBS, and the labeling effect was observed under a fluorescence microscope.

### 2.5. Identification of PGCs

RT-PCR method: The PGCs were centrifuged at 2500 rpm for 10 min, washed twice with PBS, and then 600 µL of RNAiso Plus (TaKaRa, Shiga, Japan) was added to extract total RNA. cDNA was generated by reverse transcription using the M5 Sprint qPCR RT kit with gDNA remover (Mei5bio, Beijing, China). Primers listed in Table 1 were used for PCR amplification following the instructions provided with the 2 × Rapid Taq Master Mix (Vazyme, Nanjing, China). The procedure was as follows: 95 °C for 5 min; followed by 30 cycles of 95 °C for 15 s, 55 °C for 10 s, and 72 °C for 15 s; and 72 °C for 5 min.

Immunofluorescence method: The PGCs were transferred to a gelatin-coated 24-well plate and incubated with FUT4 Rabbit pAb (ABclonal, Wuhan, China) and DAZL Rabbit pAb (ABclonal, Wuhan, China) following the provided instructions. The antibody dilution ratio was 1:100, Alexa Fluor 594-conjugated AffiniPure Goat Anti-Rabbit IgG (H + L) (ABclonal, Wuhan, China) was labeled, and then observed using an inverted fluorescence microscope (Leica, Wetzlar, Germany).

### 2.6. Identification of the Migration Ability of PGCs

The PGCs were transfected by electroporation with a plasmid pTomo-CMV-eGFP vector using a 250 V, 1000 µF exponential decay pulse. After electroporation, the cells were immediately transferred to a 6-well plate, and 3 mL of PGC culture medium was added. The cells were incubated for 48 h. Then, 0.5 µg/mL of puromycin was added for 48 h. Later, the concentration of puromycin gradually increased to 2 µg/mL. Inverted fluorescence microscopy confirmed that PGCs were labeled with eGFP.

EGFP-positive PGCs were collected, adjusted to 1 × 10^3^/μL, and injected into the bloodstream of recipient chick embryos at HH 13. After incubating embryos for 6 days, the gonads were removed and observed using an inverted fluorescence microscope.

### 2.7. Establishment of the PGC Line

The isolated PGCs were resuspended in 500 µL of culture medium and transferred to a 24-well plate. The culture medium was changed every 2 days. When the cells began to proliferate, they were transferred directly to a 12-well plate without centrifugation. After the cell count reached 1 × 10^5^, the cells were transferred to a 6-well plate. When the cell number reached 2 × 10^6^ cells, the PGCs were transferred to cell flasks for expansion or further experiments.

## 3. Results

### 3.1. PGCs Pass through Cell Culture Insert with Smaller Pore Sizes

**Figure 1:**
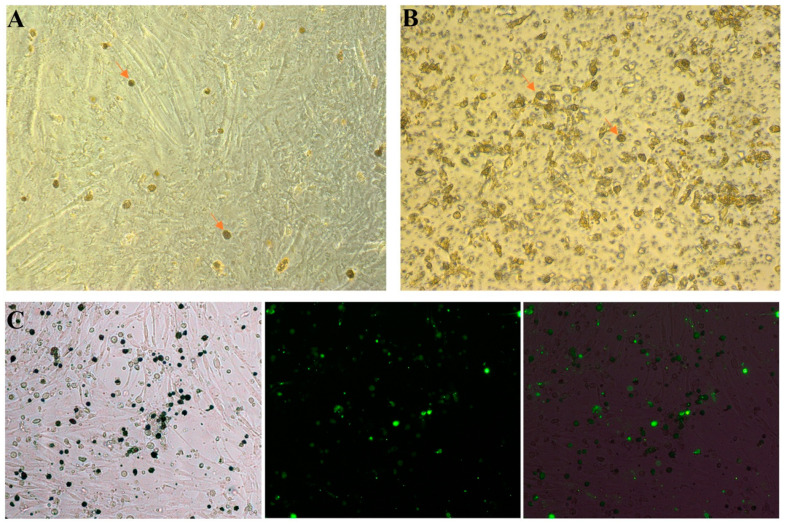
Chicken PGCs pass through 1 µm cell culture inserts. Chicken embryonic blood was placed in a 1 µm cell culture insert and co-cultured with CEFs for 3 days. (**A**) Some of the PGCs migrated to the lower layer of CEFs through the cell culture insert; (**B**) PGCs, blood cells and other impurities were retained in the insert. The orange arrow indicates PGCs. (**C**) Chicken PGCs labeled with the fluorescent dye PKH67 (green) were placed in a 1 µm cell culture insert and co-cultured with CEFs for 3 days.

Feeder cells can secrete a variety of growth factors, thereby promoting the proliferation and inhibiting the differentiation of stem cells [15,16,17]. When using a feeder layer to culture PGCs, it is possible for some feeder layer cells to become mixed in during the passage. This is not beneficial for the long-term culture of PGCs or for subsequent transgenic procedures. Studies have shown that the introduction of inserts into the feeder layer culture system can effectively reduce the interference of other cells during the passaging process [18]. Intriguingly, we used CEFs as a feeder layer and observed that certain PGCs, measuring 12–14 µm in diameter, were able to migrate to the lower CEFs using a 1 µm cell culture insert (Figure 1A). In contrast, chicken embryonic blood cells, which have a diameter of 7–8 µm, remained within the cell insert (Figure 1B). When we used CEFs at passages 3 to 5 as feeders, however, PGCs could not pass through the cell insert. To confirm that the PGCs passing through the cell culture insert did not originate from CEF cells, PGCs labeled with the fluorescent dye PKH67 (green) were placed in the 1 µm cell culture insert. As expected, the PGCs on the feeder cells showed green fluorescence, indicating that these PGCs had migrated from the upper cell culture insert (Figure 1C). Thus, we speculated that the CEF may secrete some cytokines that promote the shape change and migration of PGCs.

### 3.2. A 3 µm Cell Culture Insert Is Suitable to Isolate PGCs from Chicken Embryonic Blood

**Figure 2:**
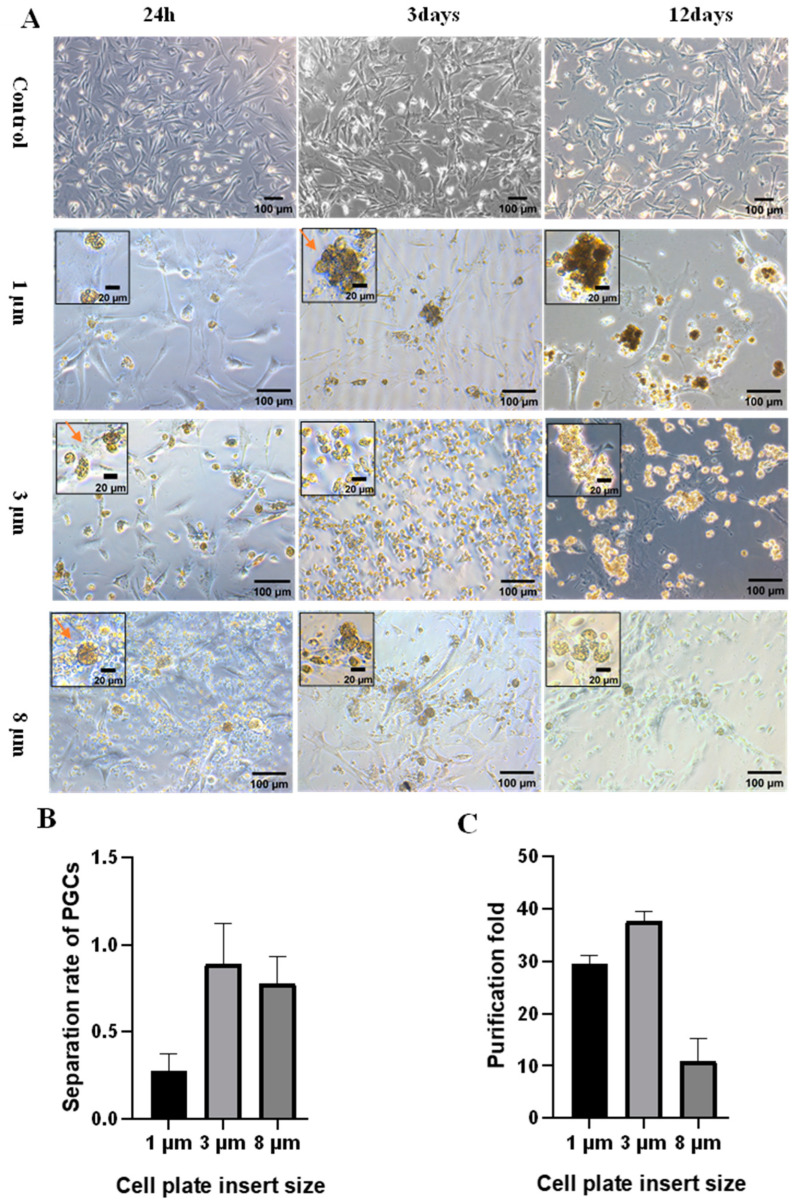
Effect of isolating PGCs using cell culture inserts with different pore sizes. (**A**) Place PGCs into three different pore size cell culture inserts and co-culture with CEFs. PGCs adhered to the lower layer of CEFs. The scale bar in the large image is 100 µm, while the scale bar in the small inset image is 20 µm. (**B**) Separation rate of PGCs using cell culture inserts. (**C**) Purity of PGCs isolated using cell culture inserts. The orange arrow shows PGCs.

Since PGCs can pass through pores smaller than their diameter, blood cells cannot. We proposed whether PGCs could be isolated from embryonic blood by utilizing this property. To test this hypothesis, we first examined the ability of PGCs to pass through cell culture inserts with various apertures. A total of 200 µL of the mixture of chicken embryonic blood and culture medium was co-transferred to cell culture inserts with pore sizes of 1 µm, 3 µm, and 8 µm, respectively. The cell culture inserts were then co-cultured with the CEFs of passages 2 to 3. After 24 h, a few PGCs passed through the 1 µm cell culture insert and adhered gently to the CEFs in the lower layer. There were no blood cells present in the lower layer. In contrast, most of the PGCs could pass through the 3 µm cell culture insert, while the blood cells were unable to pass through. For the 8 µm cell culture insert, most PGCs could pass through, but nearly half of the blood cells could also pass through (Figure 2A). Thus, we believe that the 3 µm cell culture insert is highly suitable for isolating PGCs from embryonic blood. In addition, the PGCs could be easily washed from the CEFs within 48 h after co-culture. PGCs began to proliferate after 3 days, and they were difficult to wash off from CEFs at this time. Obvious proliferating colonies were observed after 12 days of culture (Figure 2A). Blood cells were efficiently removed using a 3 µm cell culture insert, resulting in an 87.5% separation rate of PGCs (Figure 2B) and a 37.8-fold purification (Figure 2C).

### 3.3. Characteristics of PGCs Isolated from Blood Using the Cell Culture Insert/CEF Adhesion Method

**Figure 3:**
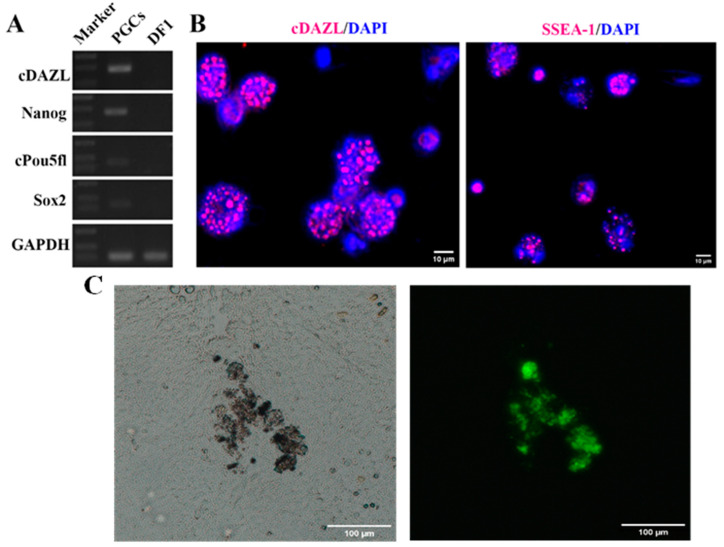
Characteristics of PGCs isolated using the cell culture insert/CEF adhesion method. (**A**) PGCs isolated using the cell culture insert/CEF adhesion method and DF1 cells were collected, and the expression of *cDAZL*, *Nanog*, *cPou5fl* and *Sox-2* was detected using RT-PCR. (**B**) The expression of *cDAZL* and *SSEA-1* in PGCs was labeled using immunofluorescence. Scale bar: 10 µm. (**C**) PGCs labeled with eGFP were injected into the egg at HH stage 13, and gonadal tissue was separated on the 6th day of incubation and compressed for observation under a fluorescence microscope. Scale bar: 100 µm.

To confirm that the cells isolated from blood using the cell culture insert/CEF adhesion method were PGCs, we utilized RT-PCR to identify the expression of PGC-specific genes. The results showed that the isolated PGCs expressed *cDAZL* (chicken azoospermia-like deletion), *Nanog*, *cPou5fl*, and *Sox-2*. However, the expression of these genes could not be detected in chicken DF1 somatic cells (Figure 3A). To further confirm the results, we also utilized immunofluorescence to detect the protein expression of *cDAZL* and *SSEA-1* (Stage-specific embryonic antigens), which are commonly employed for the identification of PGCs [15,16]. PGCs that were blown off CEFs for the first time were plated in gelatin-coated 24-well plates. After immunofluorescence cell staining, red fluorescence was observed on PGCs, but no *cDAZL* and *SSEA-1* signals were detected on CEFs (Figure 3B).

In order investigate explore whether PGCs isolated using the cell culture insert/CEF adhesion maintained their ability to migrate and colonize, the PGCs expressing eGFP were injected into eggs at HH stage 13. Two days later, green fluorescence was observed in the compressed gonadal tissue (Figure 3C), indicating that PGCs cultured without a feeder layer retained their migration ability after isolation.

### 3.4. High PGC Recovery Efficiency Achieved Using the Cell Culture Insert/CEF Adhesion Method

**Figure 4:**
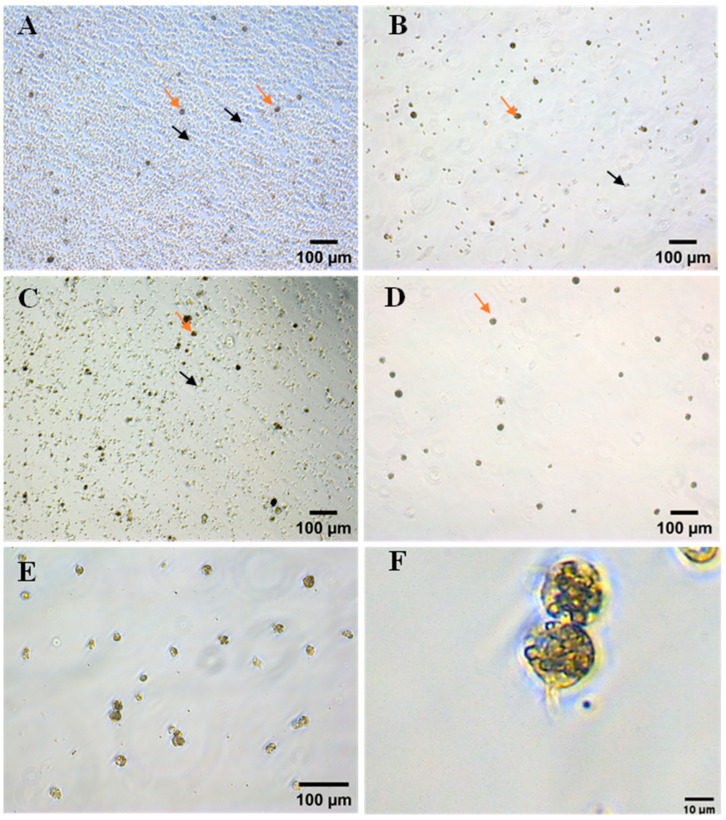
Separation of PGCs using three different separation methods. The distribution of PGCs (orange arrows) and blood cells (black arrows) in chicken embryonic blood (**A**). PGCs were separated using Percoll density gradient centrifugation (**B**), ACK lysis (**C**) and the cell culture insert/CEF adhesion method (**D**). PGCs isolated using the cell culture insert/CEF adhesion method had pseudopodia-like cytoplasmic protrusions on the surface. Scale bar: 100 µm (**E**). Magnification of the pseudopod-like cells within (**E**). Scale bar: 10 µm (**F**).

As the cell culture insert/CEF adhesion method can isolate PGCs from blood, we next tested whether this novel method had better separation efficiency than the previous PGC isolation methods, such as density gradient centrifugation and ACK lysis. Chicken embryonic blood was added to a 3 µm cell culture insert and co-cultured overnight with CEFs of passages 2 to 3. The PGCs passed through the cell culture insert and adhered to the CEFs. After gently washing the cells a few times, more than 95% of red blood cells were removed, leaving only a small amount of CEFs (Figure 4A,D) (Table 2). In contrast, 90% of blood cells were removed by Percoll density gradient centrifugation (Figure 4A,B), and the separation efficiency was 46.1% (Table 2). Lysis with ACK buffer removed 65% of red blood cells after the second lysis, resulting in a separation rate of 53.9% (Figure 4A,C) (Table 2). High-purity PGCs were obtained by removing the adherent CEFs a second time (Figure 4E). Typically, about 700 PGCs could be isolated from a single chicken embryo using the cell culture insert/CEF adhesion method. The surface of PGCs isolated using this method exhibited pseudopodia-like cytoplasmic protrusions (Figure 4F).

### 3.5. High Proliferative Potential of PGCs Isolated Using the Cell Culture Insert/CEF Adhesion Method

**Figure 5:**
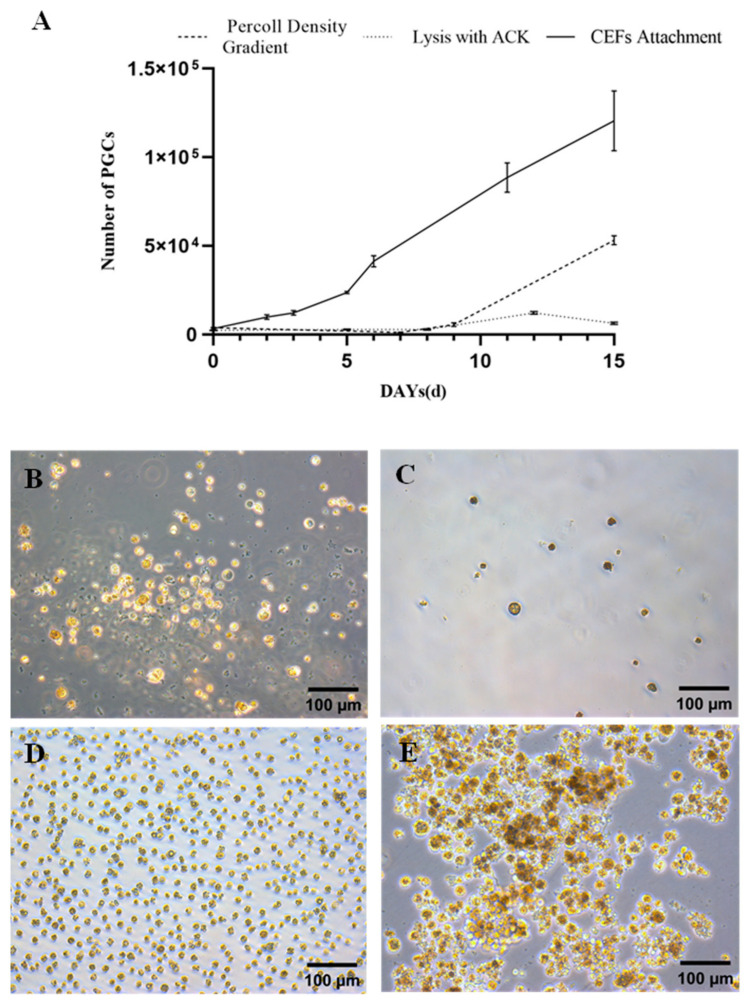
The proliferation of PGCs isolated using different methods. (**A**) The proliferation efficiency of PGCs obtained using three different separation methods. Transfer the PGCs obtained through different separation methods to a 24 well plate containing 500 µL of culture medium, continue to culture for 15 days, and then observe the condition of PGCs. Percoll density gradient centrifugation (**B**), ACK lysis (**C**), and cell culture insert/CEF adhesion method (**D**). PGCs obtained using the cell culture insertion/CEF adhesion method were cultured until day 30 (**E**). Scale bar: 100 µm.

The proliferation efficiency of PGCs is crucial for their in vitro culture. Therefore, we tested the proliferation efficiency of PGCs isolated using the cell culture insert/CEF adhesion method, as well as two other methods: Percoll density gradient centrifugation and ACK lysis. PGCs isolated using Percoll density gradient centrifugation showed partial cell apoptosis in the first few days of culture and began to proliferate on the 7th day. Within two weeks, the cells proliferated 14 times. However, some cells also died during this period, resulting in the presence of a small amount of cell debris in the culture medium. However, the remaining cells were in good condition and continued to proliferate (Figure 5A,B). The PGCs isolated by lysis with ACK buffer began to proliferate on the 9th day and underwent a 5-fold increase in proliferation by the 12th day. After 15 days, the blood cells were almost completely removed, but some cells began to differentiate, and the cells were in a state of severe shrinkage (Figure 5A,C). In contrast, PGCs isolated using the cell culture insert/CEF adhesion method started proliferating on day 2 and underwent a 37-fold increase in proliferation within two weeks (Figure 5A). The cells were in good condition and continued to proliferate after 30 days of culture, which could be directly used in subsequent experiments (Figure 5D,E).

## 4. Discussion

**Figure 6:**
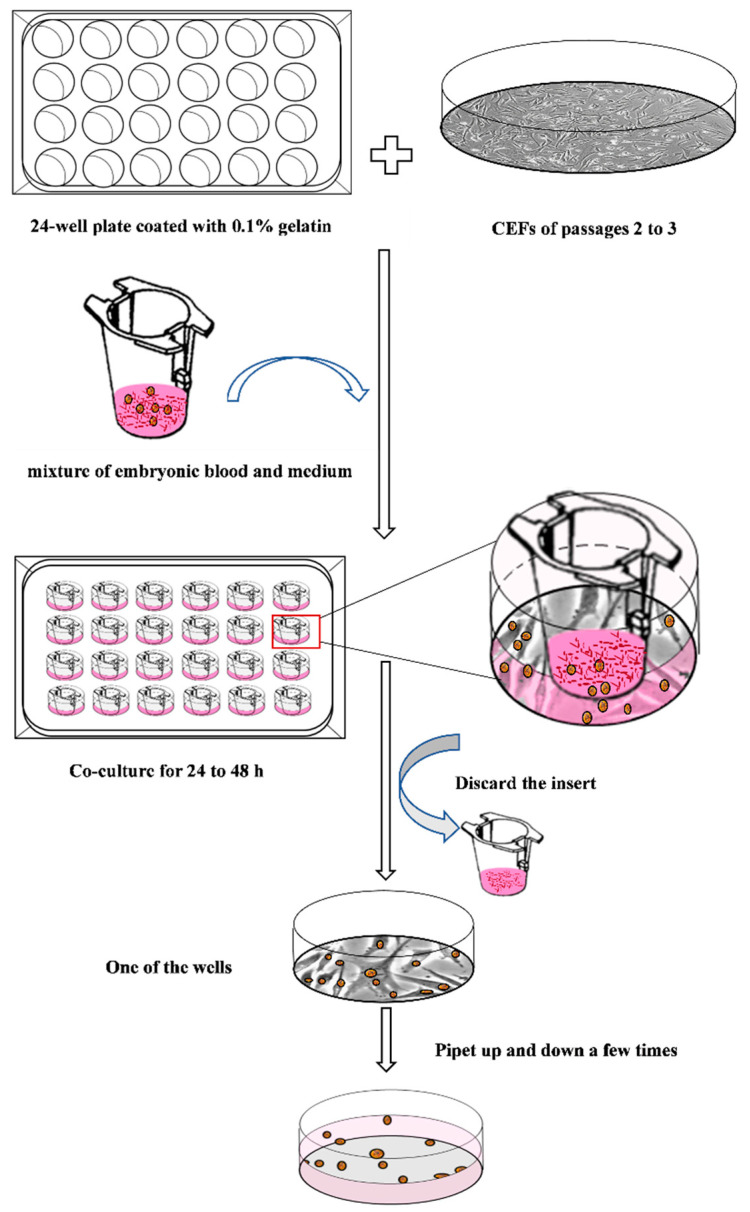
Schematic diagram of the cell culture insert/CEF adhesion method.

In this paper, a simple method was developed to isolate PGCs from chicken embryonic blood with high efficiency and promote rapid proliferation in a short period of time (Figure 6). Compared to the common methods of Percoll density gradient centrifugation and ACK lysis, this method requires a smaller sample size and offers a higher separation rate and recovery efficiency (Table 2). The isolated PGCs expressed *SSEA-1* and *cDAZL* and maintained their migration ability (Figure 3). After 30 days of culture, the cells remained in good condition and were able to continue proliferating (Figure 5).

Previous studies have shown that when using mouse embryonic fibroblasts (MEFs) as the feeder layer and adding PGCs and Mko-FI medium together into a 1 µm cell culture insert, PGCs can proliferate to 106 cells after approximately 2 weeks of culture [17]. The method simplifies the passaging steps for PGCs cultured on a feeder layer. When we employed this method for PGC culture, we were surprised to find that a small number of PGCs were able to pass through the 1 µm cell culture insert and gently adhere to the lower layer of CEFs. Using a 3 µm cell culture insert will allow a large number of PGCs to migrate to the lower CEFs, while preventing the passage of red blood cells. This effectively removes blood cells from the culture. Using this method, we successfully isolated approximately 700 high-purity PGCs from a single chicken embryo. The PGCs isolated using this method can be cultured in a feeder-free system. They have the ability to proliferate up to 37 times within a two-week period, which is comparable to the proliferation efficiency of avian PGCs cultured with a feeder layer, as reported in a previous study [18]. It is known that the diameter of PGCs is greater than 10 µm, but they can pass through inserts of 1 µm and 3 µm. When we replaced the CEF feeder cells in the lower layer with Buffalo Rat Liver (BRL), we did not observe this phenomenon. Therefore, we speculate that PGCs may undergo shape changes induced by the lower layer of CEFs, allowing them to pass through holes smaller than their diameters.

Previous studies have found that the developing gonadal ridge may produce certain chemokines to induce PGCs to colonize the gonadal ridge. In mice, the expression of BMP4 in ectoderm-derived tissues may directly or indirectly influence the differentiation of PGCs and their expression of receptors for extracellular matrix proteins or adhesion molecules [19]. In addition, a previous study found that the mouse genital ridge can produce a transfer factor called TGF-β1. This factor enables PGCs to migrate along the concentration gradient of TGF-β1 [20]. It is not clear what causes PGCs to adhere to CEFs. We speculate that there may be gonadal ridge cells in the isolated CEFs that can secrete cytokines to induce the migration and colonization of PGCs. This process may cause PGCs to deform, thus allowing them to pass through pores with smaller diameters. Further experiments found that CEFs could not induce PGCs to pass through the cell culture insert after being passaged for 4 to 5 generations. This may be due to the gradual reduction of gonadal ridge cells during the passage of CEFs, resulting in an insufficient concentration of secreted cytokines. As a result, migration and colonization of PGCs are not induced. Further research is still needed to explain the detailed mechanism.

## 5. Conclusions

In conclusion, this paper proposes a simple method to isolate PGCs with higher separation and recovery efficiencies compared to traditional methods. The PGCs isolated using this method retain their stem cell characteristics and migration ability, and exhibit good proliferation efficiency. These cells can be directly utilized for subsequent transgenic experiments or for the preservation of germplasm resources.

## Figures and Tables

**Table 1:** Primer sets used for the RT-PCR analysis of PGCs.

Gene	Primer Sequence	Product Length
*cDAZL*	F: TGTTTTTAAGTGTGCGGGCG	449 bp
	R: GCTATGGAATTGCGGTGCAG	
*Nanog*	F: CACCCAGATGCCTCTCCAG	426 bp
	R: AGGGAAGCCCTGGTGAAATG	
*cPou5fl*	F: GTTGTCCGGGTCTGGTTCT	187 bp
	R: GTGGAAAGGTGGCATGTAGAC	
*Sox2*	F: GCAGAGAAAAGGGAAAAAGGA	171 bp
	R: TTTCCTAGGGAGGGGTATGAA	
*GAPDH*	F: GAGGGTAGTGAAGGCTGCTG	109 bp
	R: CATCAAAGGTGGAGGAATGG	

**Table 2:** Separation effects of three methods on PGCs.

Separation Methods	Separation Rate (%)	Efficiency of Red Blood Cell Removal (%)	Days to Double the Number of PGCs (d)
Percoll Density Gradient	46.1	90	7
Lysis with ACK Buffer	53.9	65	9
CEFs Attachment	87.5	95	2

## Data Availability

Data are contained within the article.
